# Lipase Activity among Bacteria Isolated from Amazonian Soils

**DOI:** 10.4061/2011/720194

**Published:** 2011-10-09

**Authors:** André Luis Willerding, Luiz Antonio de Oliveira, Francisco Wesen Moreira, Mariana Gomes Germano, Aloísio Freitas Chagas

**Affiliations:** ^1^Biochemistry and Molecular Biology Coordination, Amazon Biotechnology Center (CBA), Avnue Gov. Danilo Areosa, 690 Distrito Industrial, 69075351 Manaus, AM, Brazil; ^2^Soil Microbiology Laboratory, National Research Institute of Amazonia (INPA), Caixa Postal 478, 69011-970 Manaus, AM, Brazil; ^3^Soils and Plant Tissue Laboratory, Brazilian Agricultural Research Corporation (EMBRAPA-SOY), Rod. Carlos João Strass, Distrito de Warta, 86001-970 Londrina, PR, Brazil; ^4^Department of Agronomy, Federal University of Tocantins (UFTO), Rua Badeijós, Lote 07, Chácara 69/72, Zona Rural, 77402-970 Gurupi, TO, Brazil

## Abstract

The objective of this study was to select lipase-producing bacteria collected from different counties of the Amazon region. Of the 440 bacteria strains, 181 were selected for the lipase assay in qualitative tests at Petri dishes, being 75 (41%) lipase positive. The enzymatic index was determined during fifteen days at different temperatures (30°, 35°, 40°, and 45°C). The highest lipase activity was observed within 72 hours at 30°C. Twelve bacteria strains presented an index equal to or greater than the standard used like reference, demonstrating the potential of microbial resource. After the bioassay in Petri dishes, the selected bacteria strains were analyzed in quantitative tests on *p*-nitrophenyl palmitate (*p*-NPP). A group of the strains was selected for other phases of study with the use in oleaginous substrates of the Amazonian flora, aiming for the application in processes like oil biotransformation.

## 1. Introduction

Lipase (triacylglycerol acylhydrolase E.C. 3.1.1.3) has an extensive industrial application by hydrolysing acyl ester bonds from acylglycerols at the interface between oil and water, acting also in the esterification and transesterification reactions [1–3]. Lipases are produced by many microorganisms. Most commercially useful lipases are of microbial origin. The increasing tendency of its market shows the importance to search new microbial resources to produce these enzymes [4, 5]. Lipase-producing microorganisms have been found in different habitats such as industrial wastes, vegetable oil processing factories, dairies, soil contaminated with oil, oilseeds, and decaying food [6]. Lipase from different microorganisms presents different chemical characteristics, which may be useful for industries. A specific lipase activity of *P. fluorescens* S1K W1 was found in a medium which contained emulsified olive oil as carbon source. The enzyme showed a high lipolytic activity towards tricaproic (C_6_) and tricaprylin (C_8_) compared to the other triacylglycerols examined; also, it preferentially hydrolyzed the ester bonds in 1 and 3 of triolein [7]. Temperature and pH activity also may be different among lipases [8]. 

The environmental conditions of Amazon favor high metabolic activities and growth of several microorganisms. This microbial diversity may be useful to find new good enzymatic resources. The aim of this study was to evaluate the production of lipase from the collection of soil microorganisms from the National Institute for Amazon Research (INPA), aiming for the application in processes like oil biotransformation.

## 2. Materials and Methods

### 2.1. Organisms, Maintenance, and Culture Condition

The bacterial strains were obtained from the Soil Microorganisms Collection from the National Research Institute of Amazonia (INPA). The microorganisms were isolated from soils and roots of agricultural and forest environment as phosphate-solubilizing bacteria at specific phosphatase medium [9] in a research looking for this bacterial ability to be applied in low available phosphorus Amazonian soils. Stock cultures were started in basic medium containing glucose (1.0%), yeast extract (0.2%), KH_2_PO_4 _    (0.5%), MgSO_4_    (0.2%), CaCl_2 _    (0.1%), and agar (1.8%), pH 6.5, and incubated at 30°C. For this study, 440 phosphate-solubilizing bacteria strains were tested for lipase activity. Strains that grew up to 72 hours were selected for lipase assay.

### 2.2. Lipase Production

The enzyme production was in an inducing medium (pH 8.0) containing olive oil (2.0% v/v), Tween 80 (1.0% v/v) and Rhodamine B (0.001% w/v), which makes easy the detection of the enzymatic activity, visualized by a halo close around the colonies [10–12]. Additionally, each Petri dish was subjected to UV irradiation (UV-A, 350 *η*m) mark model BIO-RAD UV Lamp 1660500. The enzymatic activity was measured by lipase activity index (LAI), a rate between the halo diameter close to the colony and the diameter of the colony during 15 days [7, 13, 14], with five replicates. The activity was measured at four temperatures (30°, 35°, 40°, and 45°C).

### 2.3. Quantitative Lipase Assay

The quantitative analysis was performed with bacterial strains that grew in basic medium up to 72 hours and incubated at 160 rpm for 48 h at 30°C in shaken flasks. After 72 hours, an aliquot (1000 *μ*L) was transferred to 49 mL of the inducing liquid medium. After 72 hours, an aliquot (2000 *μ*L) was centrifuged at 12000 g for 20 min at 4°C. The supernatant was used as the enzymatic crude extract. The activity was determined by *p*-nitrophenylpalmitate (*p*-NPP) hydrolysis by spectrophotometer model UV Mini 1240 Shimadzu at 410 *η*m [15]. An aliquot (50 *μ*L) from crude extract of each strain selected was added in 950 *μ*L of *p*-NPP solution containing 0.189 g of the substrate in 200 mL sodium acetate buffer (pH 8; 50 mM) added with 2,1% Triton X-100 [16]. The effect of temperatures was studied (from 25 to 55°C). The lipase activity was determined by the rate of *p*-nitrophenol production (*p*-NP). One unit of enzyme activity was defined as the amount of lipase required to release one *μ*mol of the *p*-NP·min^−1^.

## 3. Results and Discussion

From the 440 strains tested in the basic medium, 181 were chosen for testing lipase activity, since they presented a good preliminary development in that medium (up to 72 hours). From these, 75 strains (41%) showed enzymatic activities (Table 1).

**Figure 1:**
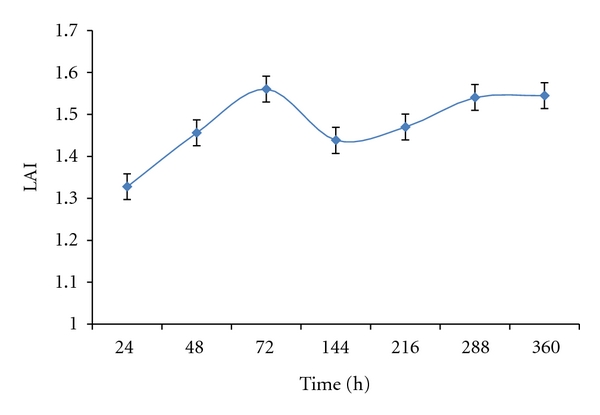
Evolution of the lipase activity index (LAI) in Petri dishes at 30°C (average of the 75 strains).

The ability of microorganisms to produce enzyme is influenced by environmental conditions such as temperature, pH, and presence of inductors or repressors. Many experiments reported in the literature showed different times for analysis, varying from 1 hour to 120 hours in lipase qualitative assay [6, 7, 13, 14]. Most of the bacteria tested in this study started lipase synthesis within 24 hours, but it was possible to observe the increase of the quantitative lipase activity until 72 hours, when occurred a declining subsequently (Figure 1). These data corroborate with the literature, indicating this time for selecting bacteria. 

**Figure 2:**
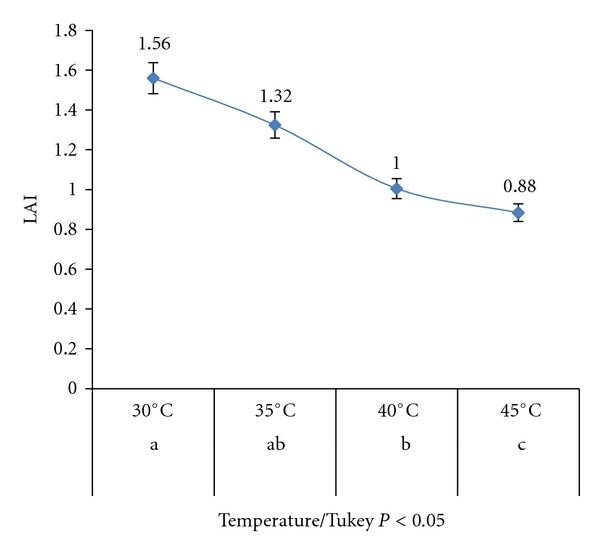
Lipase activity index (LAI) at different temperatures (average of the 75 strains).

**Figure 3:**
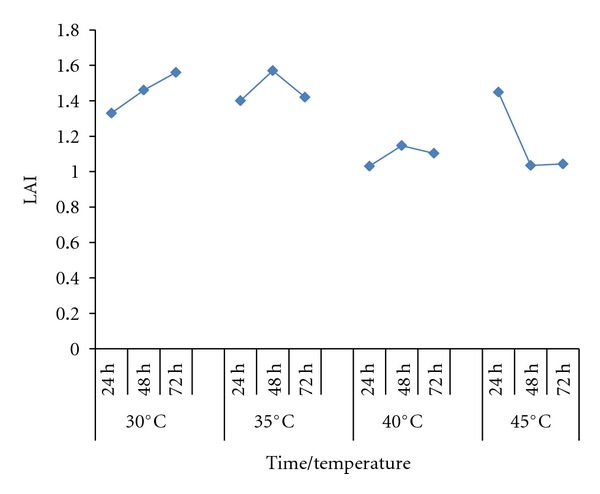
Lipase activity index (LAI) up to 72 hours at different temperatures (average of the 75 strains).

The LAI analysed in each temperature at 72 hours decreased with the increase of temperature (Figure 2). Some bacteria had a different behavior but prevailed the highest averages at 30°C. At this temperature, LAI increased linearly with the time, while, at higher temperatures, occurred a decline of the LAI values during the time of evaluation, with 48 hours being the best at 35°C and 45°C and 24 hours at 45°C (Figure 3).

In an overview, bacteria presented more lipase activity in lower temperatures, perhaps because their metabolisms accelerate when temperatures increases, resulting in depletions of enzymes activities faster. At 40°C, the lipase production presents low variation, while, at 45°C, the highest LAI occurred at 24 hours, with a strong decline after that. 

This result is similar to the observed by Dong et al. [17] with *Pseudomonas*, which presented optimum lipase activity to *Pseudomonas* at 30°C for 72 hours at pH range 7–9. However, with *Burkholderia* sp., lipase activity was analyzed at a temperature range of 25–65°C, and the highest lipase activity was obtained when the reaction was conducted at 55°C [18]. Liu et al. [18] observed that the lipase response decreased when temperature increased, remaining 96, 92, 90, and 78% of its original activity after being incubated at 25, 37, 42,5, and 55°C, respectively. This suggests that the *Burkholderia* was fairly stable during the temperature range examined. The lipases tested in this work appear to follow the same behavior.

When the application of microorganisms is wanted for industrial processes, the mechanisms of selection become important [4]. So, the analyzed parameters are decisive in screening. The highest average of the LAI happened at 30°C at 72 hours. The results corroborate with others reported, where the best lipase production occurred at 30°C [3, 6, 19, 20]. The lipase activity from *Bacillus megaterium * was analysed by Lima et al. [21] in the temperatures from 30 to 85°C, with the peak occurring between 55°C and 65°C, but they obtained good results at 29°C. Snellman et al. [27], studying an extracellular lipase of *Acinetobacter* in *p*-NPP, observed a peak of activity at 55°C, with the enzyme remaining activating up to 70°C. The optimum temperature of reaction presented in this work is higher than the reported for many bacterial lipases under similar conditions' assay. Sharma et al. [6], studying the environmental influence on lipase production, observed that *Lactobacillus plantarum* had differentiated behaviour according to the change of temperature, where the lipase activity was analyzed from 22°C to 45°C, with peak at 30°C. Sant'Anna Jr. et al. [22] tested the productivity of the enzyme at 25°C, 30°C, and 35°C, with the best results at 30°C. Stamford et al. [23] presented a process of screening lipolytic bacteria *in vitro* at 28°C. George et al. [24] presented the best results at 37°C. Sharma et al. [6] describe several works that present the best temperatures for the enzyme varying from 30°C to 60°C. 

**Figure 4:**
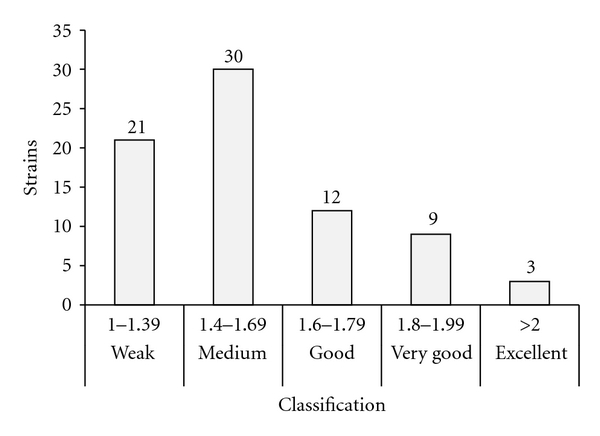
Classification of lipase activity index (LAI) for 75 strains after 72 h at 30°C in Petri dishes (olive culture).

After the definition of the ideal time and temperature, a classification was applied for the qualifying index, which revealed different levels of enzymatic activity, and it considers as reference, the strain *Bacillus subtilis* ATCC 6633 [23], that has a lipase enzymatic index of 1.80 (Figure 4). So, twelve bacteria presented a higher or similar index to the ATCC 6633. Nine bacteria were classified as “very good” and three as “excellent”, which demonstrates the potential of the studied microbiota. However, all the bacteria with enzymatic index 1.60 and a high LAI were selected to quantitative tests, totalizing 24 bacteria strains. This screening took into account that bacteria which presented a medium classification in the tests in solid medium can present better results on *p*-NPP.

**Figure 5:**
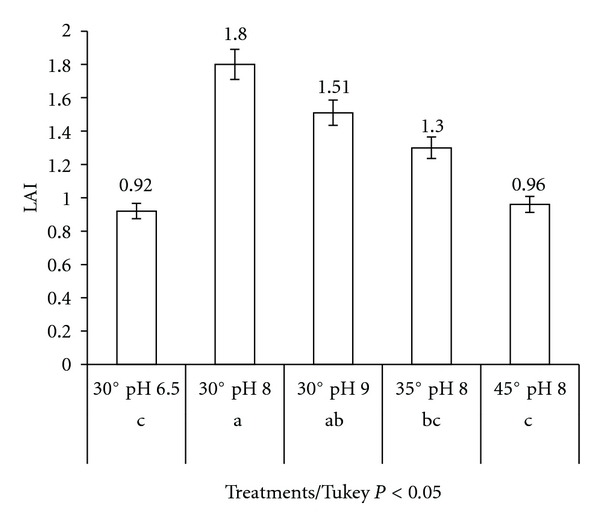
Classification of lipase activity index (LAI) for 25 strains at different treatments in Petri dishes (olive culture).

The behavior of 24 bacteria in the bioassay with different temperatures and pHs was similar to the average obtained with the 75 bacteria strains (Figure 5); there was also a highest activity at 30°C and pH 8.0. This information is important for lipase kinetic studies, when they must be tested in large scales of fermentation. The respective enzymatic indices for the 24 bacteria selected with LAI > 1.60 are in Table 2.

### 3.1. Lipase Activity

The lipases present different affinity for the substrate [24–26]. The activity varies in accordance with the chain length of the fat acids. So, some bacteria can present affinity to *p*-nitrophenylacetate (C_2_ small chains) or *p*-nitrophenycaprilate (C_8_ medium chains). Other lipase isoforms present a better affinity for esters with long carbon chain like *p*-nitrophenylaurate (C_12_) or *p*-nitrophenylpalmitate (C_16_) [6, 7, 17].

**Figure 6:**
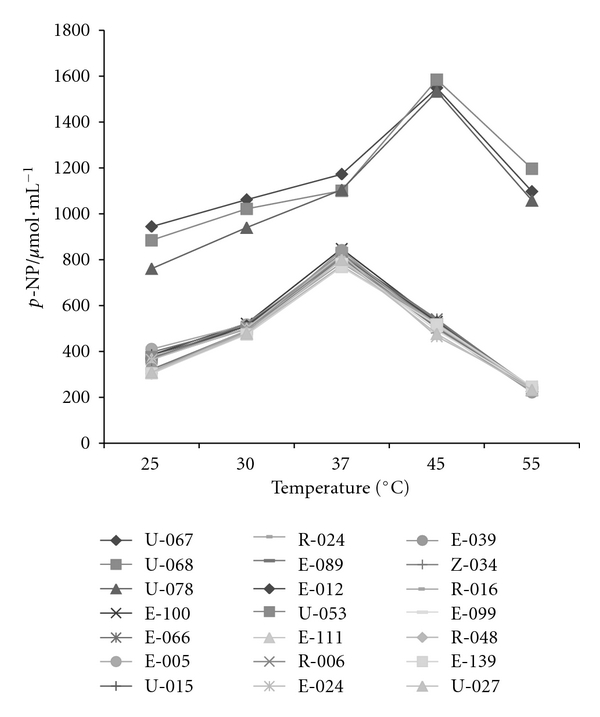
Effect of the temperature on lipase activity from crude extract on *p*-nitrophenylpalmitate (behavior of the 24 strains).

The lipase activity of the crude extracts solution of each strain selected was analyzed under *p*-NPP at different temperatures (Figure 6). The best lipase activity in the crude extract was at 45°C for the strains U-067, U-068, and U-078. 

For all bacteria, the peak of activity occurred at 37°C. These three bacteria were always superior to the rest in all test conditions. This suggests the highest affinity by lipase from these bacteria on the substrate *p*-NPP. When the peaks of the enzymatic activity are compared, the difference between the outstanding bacteria and other bacteria, at 37°C, is the double. At 45°C, the results become even 4 times bigger. The bacteria strains with the best results in this phase of the work were not those with the best results in the qualitative tests, where the analysis happened under agar medium with olive oil. Physical differences at these conditions can influence the lipase liberation. Even among the bacteria strains can be found isoforms enzymes that are differentiated by molecular weight, for example, and this may result in variation of migration in agar, but in broth culture these difficulties do not happen [27]. Though both substrates have long chains of carbon, oleic acid (C_18_), the principal component of the olive oil, and *p*-NPP (C_16_) are not the same thing. So, it is noticed that, for some bacteria, the lipase presented high affinity with the oil for others, the affinity was better with the *p*-NPP. Though they are in different assay conditions, the results presented the potential of the strains studied and indicate the group of 24 bacteria strains that was selected for other phases of study using oleaginous substrates of the Amazonian flora, aiming for the application in processes as oil biotransformation.

## Figures and Tables

**Table 1:** Amount of lipolytic bacteria for each group analyzed.

Origin (city, state)	Group	No. of strains	Activated strains	Lipase positive	%
(1) Acre (several towns)	A	4	1	1	100%
(2) Barreirinha (AM)	B	19	8	5	63%
(3) Rondônia (several towns)	D	11	2	0	0%
(4) Rio Preto da Eva (AM)	E	196	79	39	49%
(5) Manaus (Brasileirinho)	M	22	4	2	50%
(6) Projeto RECA (RO)	R	61	36	17	47%
(7) Urucu (AM)	U	86	42	10	24%
(8) Amazonas (several towns)	Z	41	9	1	11%

	Total	440	181	75	41%

**Table 2:** Lipase activity index from selected strains on olive culture medium.

Origin	Bacteria strains	Average	SD	Tukey (*P* < 0.05)
(1) INPA P-146	E-089	2.41	0.05	a
(2) INPA P-784	U-053	2.20	0.32	ab
(3) INPA P-697	U-015	2.15	0.29	abc
(4) INPA P-632	R-024	1.98	0.21	abcd
(5) INPA P-616	R-016	1.95	0.16	abcd
(6) INPA P-613	R-048	1.97	0.26	abcd
(7) INPA P-691	U-027	1.93	0.20	abcd
(8) INPA P-124	E-170	1.89	0.18	bcd
(9) INPA P-112	E-005	1.81	0.13	bcd
(10) INPA P-108	E-100	1.81	0.15	bcd
(11) INPA P-493	E-139	1.80	0.25	bcd
(12) INPA P-799	U-068	1.80	0.28	bcd
(13) INPA P-348	Z-034	1.77	0.12	bcd
(14) INPA P-478	E-066	1.71	0.19	bcd
(15) INPA P-423	E-039	1.72	0.35	bcd
(16) INPA P-803	U-078	1.69	0.19	cd
(17) INPA P-798	U-067	1.68	0.12	cd
(18) INPA P-093	E-024	1.68	0.27	cd
(19) INPA P-082	E-111	1.66	0.25	cd
(20) INPA P-117	E-012	1.63	0.12	d
(21) INPA P-540	E-087	1.62	0.09	d
(22) INPA P-106	E-099	1.62	0.22	d
(23) INPA P-392	E-053	1.61	0.17	d
(24) INPA P-593	R-006	1.60	0.12	d
